# BMP receptor 2 inhibition regulates mitochondrial bioenergetics to induce synergistic cell death with BCL-2 inhibitors in leukemia and NSLC cells

**DOI:** 10.21203/rs.3.rs-5065904/v1

**Published:** 2024-09-12

**Authors:** Ashley Toussaint, Manohar Singh, Guoquiang Wang, Monica Driscoll, Vrushank Bhatt, Jean De La Croix Ndong, Sahil Shuaib, Harrison Zoltowski, John Gilleran, Youyi Peng, Anastassiia Tsymbal, Dongxuan Jia, Jacques Roberge, Hellen Chiou, Jessie Yanxiang Guo, Daniel Herranz, John Langenfeld

**Affiliations:** Rutgers State University of New Jersey; Rutgers State University of New Jersey; Rutgers State University of New Jersey; Rutgers State University of New Jersey; Rutgers Cancer Institute of New Jersey; Rutgers State University of New Jersey; Rutgers State University of New Jersey; Rutgers State University of New Jersey; Rutgers State University of New Jersey; Rutgers Cancer Institute of New Jersey; Rutgers State University of New Jersey; Rutgers State University of New Jersey; Rutgers State University of New Jersey; Rutgers State University of New Jersey; Rutgers Cancer Institute of New Jersey; Rutgers Cancer Institute of New Jersey; Rutgers State University of New Jersey

**Keywords:** BMP inhibitor, cell death, mitochondrial calcium, cancer, free radicals

## Abstract

**Background:**

Bone morphogenetic protein (BMP) signaling cascade is a phylogenetically conserved stem cell regulator that is aberrantly expressed in non-small cell lung cancer (NSLC) and leukemias. BMP signaling negatively regulates mitochondrial bioenergetics in lung cancer cells. The impact of inhibiting BMP signaling on mitochondrial bioenergetics and the effect this has on the survival of NSLC and leukemia cells are not known.

**Methods:**

Utilizing the BMP type 2 receptor (BMPR2) JL189, BMPR2 knockout (KO) in cancer cells, and BMP loss of function mutants in *C elegans*, we determined the effects of BMPR2 inhibition (BMPR2i) on TCA cycle metabolic intermediates, mitochondrial respiration, and the regulation of mitochondrial superoxide anion (SOA) and Ca^++^ levels. We also examined whether BMPR2i altered the threshold cancer therapeutics induce cell death in NSLC and leukemia cell lines. KO of the mitochondria uniporter (MCU) was used to determine the mechanism BMPR2i regulates the uptake of Ca^++^ into the mitochondria, mitochondrial bioenergetics, and cell death.

**Results:**

BMPR2i increases mtCa^++^ levels and enhances mitochondrial bioenergetics in both NSLC and leukemia cell lines that is conserved in C elegans. BMPR2i induced increase in mtCa^++^ levels is regulated through the MCU, effecting mitochondria mass and cell survival. BMPR2i synergistically induced cell death when combined with BCL-2 inhibitors or microtubule targeting agents in both NSLC and leukemia cells. Cell death is caused by synergistic increase in mitochondrial ROS and Ca^++^ levels. BMPR2i enhances Ca^++^ uptake into the mitochondria induced by reactive oxygen species (ROS) produced by cancer therapeutics. Both acute myeloid leukemia (AML) and T-cell lymphoblastic leukemia cells lines were more responsive to the JL189 alone and when combined with venetoclax or navitoclax compared to NSLC.

## Background

Bone morphogenetic protein (BMP) signaling, which is phylogenetically conserved, regulates cell fate decisions throughout embryonic development. BMP signaling is aberrantly expressed in NSLC and AML [1, 2]. There are more than 20 BMP ligands that signal through type 1 (Alk2, Alk3, Alk6) receptors (BMPR1) and type 2 (BMPR2A, ACVR2A, and ACVR2B) serine-threonine kinase receptors. In the canonical BMP pathway, ligand binding to BMPR1 promotes phosphorylation by constitutively active BMPR2, leading to the activation of the Smad-1/5 transcription factor [3]. The noncanonical BMPR2 pathway signals independently of Smad-1/5 [4]. Smad-1/5-dependent signaling includes the transcriptional activation of inhibitor of differentiation proteins (ID1–3) [5, 6], which regulate cancer cell metastasis and stemness of cancer stem cells [7]. The noncanonical BMPR2 pathway induces the expression of the potent antiapoptotic proteins X-linked inhibitor of apoptosis (XIAP) [8], TGFß activated kinase (TAK1), PI3K, and microtubules (MTs) independent of BMPR1 [9].

BMP signaling suppresses the master regulator of AMP-activated kinase (AMPK) catabolism during nutrient stress in NSCLC cell lines, which is conserved in *C. elegans* [10]. AMPK increases mitochondrial biogenesis [11, 12] and increases mitochondrial respiration by increasing Ca^++^ uptake through the mitochondrial uniporter [13]. BMPR2 signaling promotes anabolic metabolism in lung cancer cells and *C. elegans* by activating PI3K/Akt/mTOR signaling [10, 14, 15]. Interestingly, the BMP2 ligand causes a rapid decrease in tricarboxylic acid cycle (TCA) intermediates and nonessential amino acids in NSLC cells [10]. These studies suggest that BMP signaling negatively affects mitochondrial bioenergetics. BMPR2 inhibition (BMPR2i) synergistically induces mitochondria-induced cell death when combined with YM155 or TRAIL [16–18] in NSLC cells. The mechanisms by which BMPR2i synergistically mediates mitochondrial induced cell death and its effects on mitochondrial bioenergetics are poorly understood. Leukemias are more dependent on oxidative phosphorylation for ATP production compared to NSLC so could respond differently to BMPR2i. The effects of BMPR2i on the survival and mitochondrial bioenergetics in leukemia cells is not known.

We show here that BMPR2i increases mitochondrial bioenergetics, mtCa^++^ and mtROS levels in both NSLC and leukemia cells. Our studies suggest that BMPR2i regulates mitochondrial redox sensing that promotes mtROS and mtCa^++^ overload and cell death when combined with cancer therapeutics that increase ROS levels. Leukemia cells are more responsive to BMPR2 inhibition induced increase in mtCa^++^ levels and cell death compared NSLC cells. Our studies suggest that a BMPR2 inhibitor may be effective in treating AML with p53 and MLL translocations, which are resistant to chemotherapeutics.

## Materials and Methods

### Chemicals and reagents

JL189 was designed and synthesized by John Gilleran, Anastasiia Tsymbal, and Jacques Roberge from Rutgers Molecular Design and Synthesis [17]. Human recombinant BMP-2 was purchased from R&D Systems. MitoTracker green, Rhod-2AM, Fluo-4AM, TMRM, Cell ROX Green, and MitoSox Red were purchased from Invitrogen (Waltham, MA, USA). ABT 263, vitamin E, apocynin, cisplatin, and buthionine sulfoximine (BSO) were purchased from Med Chem Express (NJ, USA).

### Antibodies

Phospho-Smad1/5, Smad1, ID1, AIF, Smac/DIABLO, cytochrome c, mtATP8, TFAM and cleaved caspase 3 rabbit monoclonal antibodies were purchased from Cell Signaling Technology (Beverly, MA, USA). PARP1 and B-actin were purchased from BD Biosciences (San Jose, CA, USA) and Sigma (St. Louis, MO, USA), respectively.

### Cell viability analysis

Cells were seeded in 6-well plates and treated with selected agents. After the predetermined time point, live and dead cells were counted by a Vi-CELL BLU cell viability analyzer (Beckman Coulter Brea, CA, USA), which stains cells with trypan blue. The combination index (CI) was calculated using Compusyn software, and the IC_50_ was calculated using GraphPad Prism.

### Cell culture

The human cell lines H1299, Calu-1, and HEK-231 were purchased from American Type Culture Collection (ATCC) (Manassas, VA, USA). Jurkat and MDA-MB231 WT and MCU KO cells were generous gifts from Mohamed Trebak April of 2024 [19] (University of Pittsburgh) and Cristina Mammucari [20] (University of Padova, Italy), respectively. Jurkat and MDA-MB 231 cells were maintained in RPMI-1640 culture medium supplemented with 10% FBS. H1299, A549 WT, and A549 BMPR2 KO cells were maintained in low-glucose DMEM supplemented with 5% FBS, McCoy’s medium supplemented with 10% FBS (Calu-1) and high-glucose DMEM supplemented with 10% FBS (HT-22) in a humidified incubator at 37°C with 5% CO_2_. All media were supplemented with 1× antibiotic and 1× L-glutamine. A549 BMPR2 KO and A549 WT cells with a 104 bp deletion of exon 11 in the kinase domain were purchased from FenicsBio (Helethorpe, MD) November 2023. HAP-1 WT and VDAC1 KO cells were purchased from Horizon Discovery on October 2023. HT-22 cells were purchased from Millipore October 2022 (October 2022). Cell lines were authenticated by suppliers and donors.

### Metabolomics

LC − MS analysis of the cellular metabolites was performed on a Q Exactive PLUS hybrid quadrupole-orbitrap mass spectrometer (Thermo Scientific) coupled to hydrophilic interaction chromatography (HILIC) as previously reported [21, 22].

### Immunofluorescence imaging

Immunofluorescence was performed as previously described [17]. In brief, cells were plated onto sterile coverslips and treated for the designated time points. The cells were stained with MitoTracker Green for 30 minutes. The cells were washed and counterstained with DAPI. The cells were examined using a 60X oil lens and a fluorescence microscope (Nikon Eclipse TE300). The studies were performed at least 4 times.

### TMRM Staining

The cells were treated with DMSO (control), selected agents, and 50 μM CCCP (+ ve control) and subsequently stained with TMRM (Thermo-M20036) for 30 minutes at 37°C. After staining, the cells were washed with PBS, and after 30 minutes, the data were acquired on a Cytek Aurora flow cytometer.

### Cytoplasmic and mitochondrial calcium analysis by flow cytometry

Mitochondrial and cytoplasmic calcium were analyzed using Rhod-2AM and Fluo-4AM staining, respectively. In brief, equal amounts of Flu-4AM or Rhod-2AM stock solution and the nonionic detergent Pluronic F-127 were diluted in media. The cells were stained for 45 minutes at 37°C. After staining, the cells were washed with PBS, and the data were acquired on a Cytek Aurora flow cytometer.

### Apoptosis analysis by Annexin-FITC staining and flow cytometry

Apoptosis was analyzed by flow cytometry using a FITC Annexin V Apoptosis Detection Kit I (BD, Biosciences 559763) according to the manufacturer’s protocol. In brief, cells were treated for the designated time points. After treatment, the cells were washed and stained with 100 μl of staining solution (5 μl of Annexin V-FITC and 5 μl of 7-AAD in 1X binding buffer). After staining, 400 μl of 1X binding buffer was added to each tube, and the cells were analyzed for apoptosis via flow cytometry (Cytek Aurora).

### MitoTracker Green analysis by flow cytometry

The cells were treated with DMSO (control) and selected agents and subsequently stained with MitoTracker Green for 30 minutes at 37°C. After staining, the cells were washed with PBS, and the data were acquired on a Cytek Aurora flow cytometer.

### Western blotting

Western blotting was performed as previously described [17]. In brief, total protein was isolated by RIPA lysis buffer, subjected to SDS‒PAGE and transferred to nitrocellulose membranes. The membrane was incubated with primary antibodies overnight at 4°C, followed by incubation with HRP-conjugated secondary antibodies for 1 hour at room temperature. The band was visualized on X-ray films.

### Lung cancer xenograft

H1299 cells were mixed with 50% Matrigel in PBS, and 2×10^6^ cells were injected into the flanks of NCr nude mice (Taconic Biosciences). Tumors were isolated, and a single-cell suspension was prepared. A total of 1×10^6^ cells were injected subcutaneously into the mice with 50% Matrigel. After visible tumor development, the mice were randomized into vehicle, JL189, ABT 263, and JL189 + ABT263 groups. JL189 was dissolved in 5% NMP, 5% Solutol HS-15 and 90% citric acid, and ABT263 was dissolved in 30% PPG, 5% T80, 3.30% D5W, and 1% DMSO. JL189 (30 mg/kg twice a day) and ABT263 (12 mg/kg once a day) were injected by intraperitoneal (IP) injection 5 days a week for 3 weeks. Tumor size and volume were measured. Mice were euthanized for tumors exceeding 15 mm × 15 mm, pain, or loss of 20% body weight, as approved by Rutgers IACUC regulations, which were not met during the study.

### Pharmacokinetics

The pharmacokinetic properties of JL189 were examined in BALB/c mice following intraperitoneal injection as previously described [23](Sai Life Science, Pune India).

### C elegans

#### Strains and maintenance

We maintained all *C. elegans* strains on nematode growth media (NGM) supplemented with OP50–1 *Escherichia coli* in a 20°C incubator. We kept all animals on food for at least 10 generations before they were used in the test. The strains used in the current study included the following:

1. ZB5721: ccIs4251 [P_*myo-3*_::GFP::LacZ::NLS + P_*myo-3*_::mitochondrial GFP + dpy-20(+)] I; aceIs1 [P_*myo-3*_::mitochondrial LAR-GECO; P_*myo-2*_::RFP] II
2. ZB5708: ccIs4251 [P_*myo-3*_::GFP::LacZ::NLS + P_*myo-3*_::mitochondrial GFP + dpy-20(+)] I; aceIs1 [P_*myo-3*_::mitochondrial LAR-GECO; P_*myo-2*_::RFP] II; *dbl-1(wk70)* V

Worms were imaged with a spinning disc confocal microscope under the GFP channel or the RFP channel at the same z-stack. Images were processed and analyzed with ImageJ.

### Statistical analysis

A paired Student’s t test, assuming unequal variances, was used to compare the means of the control with the mean of each treated group. Differences with p values < 0.05 were considered to indicate statistical significance. * p < 0.05, ** p < 0.01, *** p < 0.001, **** p < 0.0001.

## Results

### BMPR2 inhibition increases TCA cycle intermediates.

**Figure 1:**
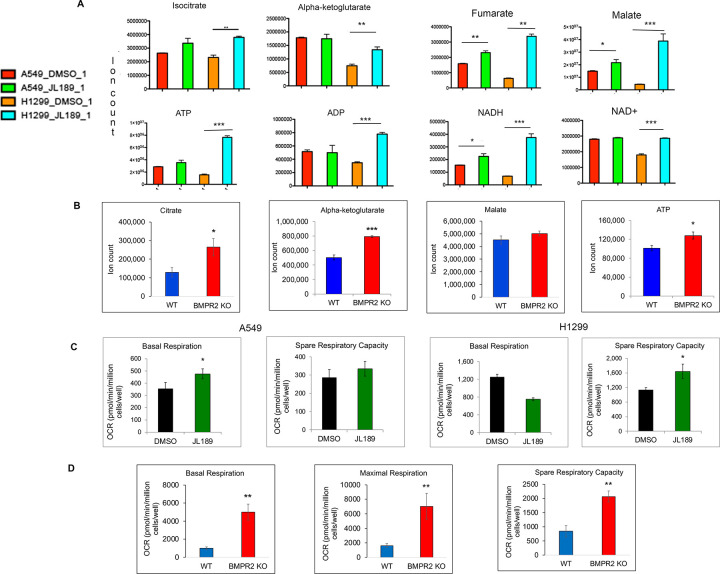
BMPR2i increases TCA cycle intermediates and mitochondrial respiration. **(A)** A549 and H1299 lung cancer cell lines were treated with JL189 2.5 mM for 24 hr and then examined for **(A)** metabolomics done by liquid chromatography-mass spectrometry (LC-MS), **(B)**Metabolomics were performed comparing TCA cycle intermediates of A549 WT and A549 BMPR2 KO cells **(C)** Mitochondrial respiration was determined by an Agilent Seahorse analyzer on A549 and H1299 cells treated with JL189 2.5 mM for 24 hr. **(D)** Comparison of mitochondrial respiration between A549WT and A549 BMPR2 KO cells. The studies were performed 3–4 times

**Figure 2:**
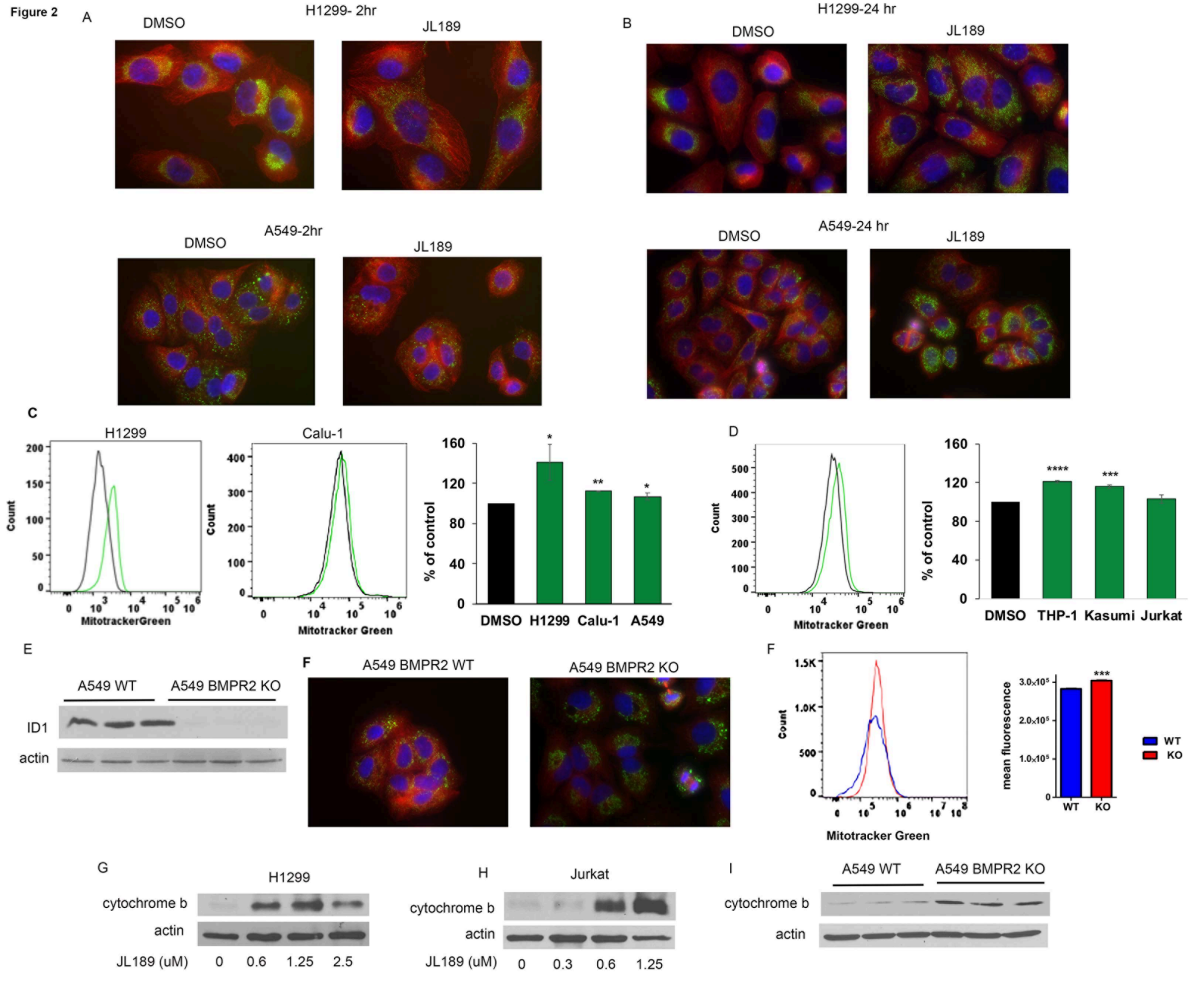
BMPR2i increases mitochondrial mass. **(A-B)** Co-immunofluorescent imaging of a-tubulin (red) and TFAM1 (green) with nucleus stained with DAPI (blue) of H1299 and A549 cells treated with JL189 2.5 mM for 2 and 24 hr. Cell treated with JL189 exhibited trafficking of the mitochondria with greater TFAM1 fluorescence after 24 hr. **(C)**MitoTracker Green fluorescence was analyzed by flow cytometry after 24 hr of treatment with JL189 2.5 mM or DMSO control. The graph represents the mean of 4 studies presented as the percentage of control. **(D)** Coimmunofluorescent imaging of a-tubulin (red) and TFAM1 (green) in A549 WT and KO cells. **(E)** MitoTracker Green analysis demonstrate that A549 BMPR2 KO cells have significantly greater fluorescence compared to A549 WT cells. The graph shows mean fluorescence of 4 studies. **(F)**Western blot analysis of total protein lysate from A549 WT and A549 BMPR2 KO cells demonstrating an increase in cytochrome b.

BMP2 ligand was previously shown to decrease TCA cycle intermediate in H1299 and A549 NSLC cell lines [17]. We used JL189 a selective inhibitor of BMPR2 [17] to determine if BMP inhibition altered expression of TCA cycle intermediates in lung cancer cell lines. JL189 significantly increased the levels of the TCA cycle intermediates isocitrate, alpha-ketoglutarate, fumarate, malate, and ATP in H1299 cells (Fig. 1A). In A549 cells, JL189 significantly increased the expression of fumarate, malate and nicotinamide adenine dinucleotide (NADH) (Fig. 1A). Western blot of A549 cells with kinase domain deletion of BMPR2 demonstrated significant decrease in ID1 compared to WT confirming downregulation of BMP signaling (Fig. 2E). A549 cells with BMPR2 KO had significantly greater levels of citrate, alpha-ketoglutarate, and ATP than A549 WT cells (Fig. 1B). To determine whether BMPR2i affects metabolism in noncancerous cells, metabolomic studies were performed on the HT-22 hippocampal neuronal mouse cell line. In HT-22 cells, JL189 decreased the levels of the glycolysis intermediates lactate and pyruvate while maintaining or increasing the levels of TCA cycle intermediates **(Fig S1)**. The malate/pyruvate and α-ketoglutarate/pyruvate ratios increased, suggesting an increase in mitochondrial respiration **(Fig S1)**. Both pharmacological and genetic inhibition of BMPR2 signaling showed similar effects on TCA cycle.

### BMPR2 inhibition increases oxidative phosphorylation.

Inhibition of BMPR2 with JL189 in A549 cells significantly increased basal respiration, while spare respiratory capacity significantly increased in H1299 cells (Fig. 1C). A549 BMPR2-KO cells had significantly greater basal respiration, maximal respiration and spare respiratory capacity compared to A549 WT cells (Fig. 1D). Together, these data support that BMPR2i increases mitochondrial respiration in NSLC cell lines.

### BMPR2 inhibition increases mitochondrial mass.

Dual immunofluorescence staining was performed for tubulin and the mitochondrial protein TFAM. Like prior studies using BMPR2 siRNA [24], inhibition of BMPR2 with JL189 destabilized the MT within 2 hr, and there was a clear change in the position of the mitochondria. In cells treated with JL189, the mitochondria moved from their typical perinuclear position to across the cytosol (Fig. 2A). By 24 hours, the intensity of TFAM fluorescence was much greater in cells treated with JL189 (Fig. 2B), suggesting that there were more mitochondria. To quantify the mitochondrial mass, the cells were loaded with MitoTracker Green and examined by flow cytometry. MitoTracker Green fluorescence was significantly greater in JL189-treated cells than in vehicle control-treated cells in NSLC cell lines H1299, Calu-1, and A549 cells (Fig. 2C). MitoTracker Green fluorescence was also significantly greater in the AML cell lines THP-1 and Kasumi-1 treated with JL189 (Fig. 2D). TFAM fluorescence was also more intense in A549 KO cells than in WT cells (Fig. 2F). Compared with A549 WT cells, A549 BMPR2 KO cells also exhibited significantly greater MitoTracker Green fluorescence (Fig. 2G). Cytochrome b is synthesized from mitochondrial DNA [25]. JL189 increased cytochrome b expression in H1299 and Jurkat cells **(Fig G-H)**. The expression of cytochrome b was greater in A549 BMPR2-KO cells compared to A549 WT cells (Fig. 2I). These data suggest that BMPR2 inhibition increases mitochondrial bioenergetics in NSLC and leukemia cell lines.

### BMPR2 inhibition increases mitochondrial calcium (mtCa ^++^ ) levels.

**Figure 3:**
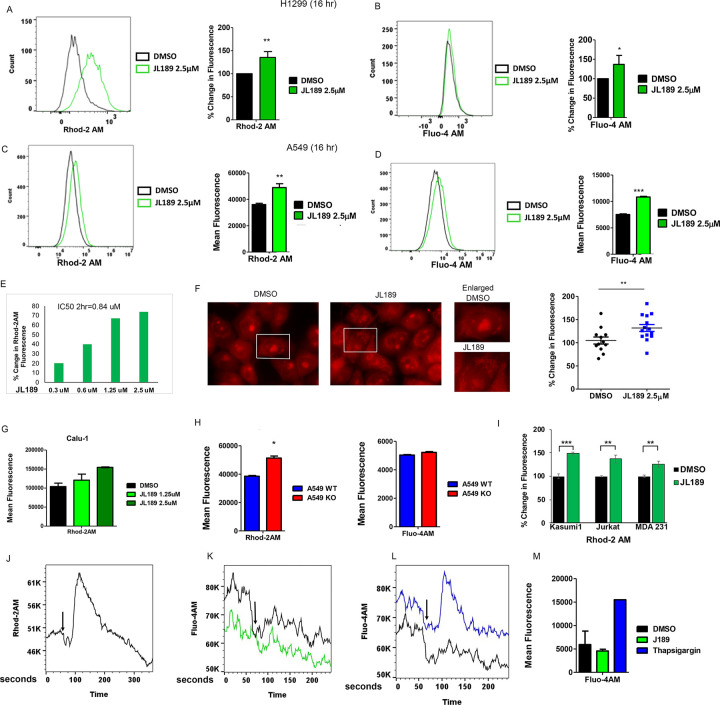
BMPR2i in lung cancer cells increases mtCa++ levels. **(A-B)** H1299 and **(C-D)** A549 cells treatment with DMSO or JL189 for 16 hr then loaded with Rhod-2AM (mitochondria Ca^++^) or Fluoro-4AM (cytosol Ca^++^) and DAPI then examined by flow cytometry. The data represents the mean % change in fluorescence or mean fluorescence of 4 experiments. **(E)** The IC_50_ of JL189 to increase Rhod2-AM fluorescence by 50% in H1299 cells treated for 2 hr. **(F)** Immunofluorescence imaging of H1299 cells loaded with Rhod2AM and then treated with JL189 2.5 mM for 2 hr. Increased fluorescence is noted in the perinuclear organelles (mitochondria) in cells treated with JL189. The Fluorescence in more that 100 cells was quantified by image J and is reported as the percent increase from baseline. **(G)** Rhod2AM fluorescence of Calu1 cells treated with JL189 for 2 hr. **(H)** Mean Rhod2-AM and Fluoro-4AM fluorescence of 3–4 studies of A549 WT and A549 BMPR2 cells. **(I)** Percent increase in Rhod2AM fluorescence of cells treated with JL189. Kasusmi1 and Jurkat cells treated with JL189 1.25 mM for 16 hr. MDA 231 treated with JL189 2.5 mM for 5 hr. Graph represents the mean of 3–4 studies **(J)** Representative flow cytometry analysis (n=5) of Calu-1 cells loaded with Rhod2AM then treated with JL189 2.5 mM without calcium for 5 minutes. **(K-M)** Calu1 cells loaded with Fluo-4AM were treated with **(K,M)** JL189 2.5 mM or **(L,M)** Thapsigargin 300 nM without calcium and analyzed by flow cytometry for 3 minutes. **(M)** The graph shows the mean Fluo-4AM fluorescence of 2 studies. Arrows mark the time of treatment.

The influx of calcium into the mitochondria through the mitochondrial calcium uniporter (MCU) is a conserved mechanism that controls mitochondrial respiration, cell survival, and cell death when levels become too high [26, 27]. Using the mitochondrial Ca^++^ indicator Rhod2AM quantified by flow cytometry, we examined whether BMPR2i regulated mtCa^++^ levels in NSLC and leukemia cells. JL189 significantly increased mtCa^++^ levels in H1299, A549, and Calu-1 RAS mutated NSLC cell lines (Fig. 3A, C, G). There was also a significant increase in cytosolic Ca^++^ levels **(Fig B, D)**. The concentration of JL189 that increased mtCa^++^ levels by 50% at 2 hr in H1299 cells was 0.84 μM (Fig. 3E). Immunofluorescence imaging confirmed that the increase in Rhod2AM fluorescence occurred within the mitochondria and not within the cytosol (Fig. 3F).

A549 BMPR2-KO cells had significantly greater mtCa^++^ levels than did A549 WT cells, confirming that BMPR2 inhibition increases mtCa^++^ levels (Fig. 3H). There was no difference in cytosolic Ca^++^ levels between the A549 WT and A549 BMPR2 KO cells (Fig. 3H), suggesting that the increase in cytosolic Ca^++^ levels induced by JL189 was a secondary event. JL189 also increased mtCa^++^ levels in leukemia cell lines, Jurkat (T-ALL) and Kasumi1 (AML) and triple negative breast cancer (TNBC) cell line MDA 231 (Fig. 3I).

### BMPR2 inhibition increases mtCa ^++^ levels in the absence of Ca^++^.

Calu1 cells treated with JL189 in calcium-free media exhibited an increase in mtCa^++^ levels within 20 seconds, which returned to baseline after 120 seconds (Fig. 3J). JL189 did not change cytosolic Ca^++^ levels after 3 minutes (Fig. 3K,M). Thapsigargin, which blocks endoplasmic Ca^++^-ATPase (SERCA) and leads to an increase in cytosolic Ca^++^, was used as a positive control (Fig. 3L–M). These studies indicate that the increase in mtCa^++^ following BMPR2 inhibition is not caused by an increase in the uptake of extracellular calcium. The studies also suggest that the increase in mtCa^++^ levels is not from an increase in cytosolic Ca^++^ levels released from calcium storage organelles.

### BMP inhibition in C. elegans increases mtCa^++^ levels and mitochondrial mass.

**Figure 4.**
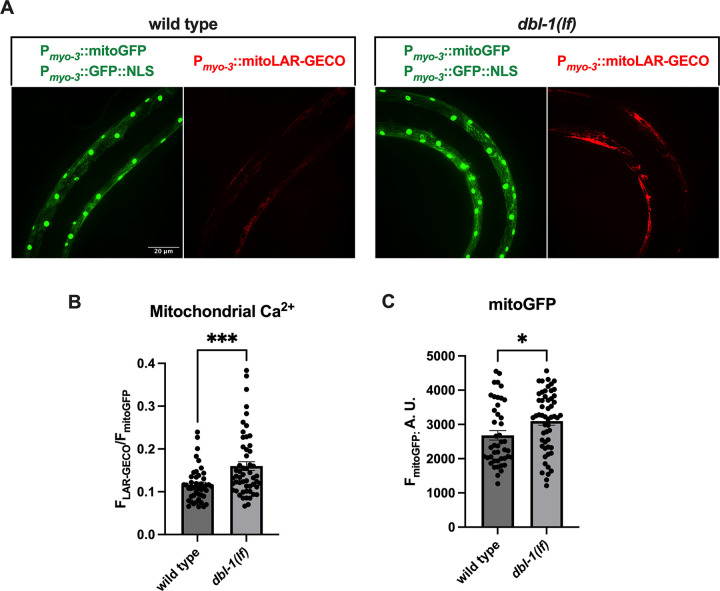
BMP inhibition in C elegans increases mtCa^++^ and mitochondrial mass. WT and dbl-1(BMP ligand) *lof* mutant worms were crossed to generate transgenes expressing the mtCa^++^ sensor LAR-GECO (red) and mitoGFP (green) under the control of the *myo-3* promoter. MitoGFP localizes to the nucleus and mitochondria in muscle and was used to normalize mtCa^++^ levels and determine mitochondrial mass. The fluorescence intensity was determined by confocal microscopy. A total of six independent trials with a n = ~50 worms were performed to determine the mtCa^++^ levels and mitochondrial mass. **(A-B)** The mean fluorescence intensities of LAR-GECO and **(A,C)** MitoGFP were significantly greater in dbl-1(BMP ligand) *lof* mutants than in WT.

To determine whether BMP regulation of mitochondrial calcium and mitochondrial mass is conserved, we utilized *C. elegans* harboring BMP ligand (dbl-1) loss-of-function (*lof*) mutants and the red fluorescent mitochondrial calcium sensor LAR-GECO [28] under the control of the *myo-3* promoter [29]. Worms were also crossed to generate animals that expressed the green fluorescent protein (GFP) transgene under the control of the *myo-3* promoter, which localizes to the mitochondria (MitoGFP) and nucleus. MitoGFP was used to determine mitochondrial mass, and the LAR-GECO fluorescence intensity was normalized to MitoGFP to determine basal mtCa^++^ levels. Compared with those of the WT, the animals harboring the dbl-1 *lof* transgene had significantly greater mtCa^++^ levels (Fig. 4A–B) and greater mitochondrial mass (Fig. 4A, C).

### BMPR2i synergistically enhances cell death when combined with BCL-2 inhibitors.

**Figure 5:**
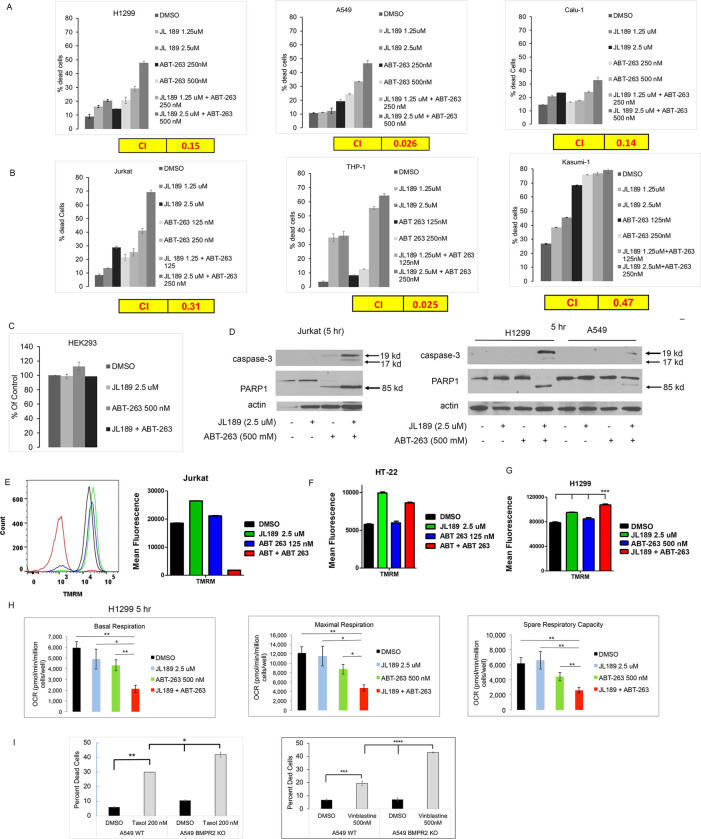
BMPR2i synergizes with ABT-263 and microtubule-targeting chemotherapeutic agents to induce mitochondrial cell death. **(A)** NSLC and **(B)** leukemia cells were treated with JL189 and ABT-263 alone or in combination with ABT-263 for 48 hours. The percentage of dead cells was determined with trypan blue staining using the Vi-CELL BLU cell viability analyzer. The combination index (CI) < 1.0 indicates synergy which is highlighted in yellow. **(C)** Cell counts of treated HEK 293 cells. Studies are representative experiments done in duplicate **(D)** Western blot analysis of cells treated for 5 hr demonstrating the expression of activated caspase-3 and cleaved PARP1 in the combination group. **(E)**Jurkat cells were treated for 2 hr and the mitochondrial membrane potential (MMP) was determined after loading the cells with TMRM and then analyzed by flow cytometry. **(F)** TMRM fluorescence of HT-22 cells treated for 5 hr. Graphs represent the mean of 2 studies. **(G)** TMRM fluorescence of H1299 cells treated for 24 hr. Graphs represent the mean of 3 studies. **(H)**Mitochondrial respiration was determined by an Agilent Seahorse analyzer of cells treated for 5 hr. The data represents the mean of 3 studies. **(I)** Cell counts of A549 WT and A549 KO cells treated with Taxol (mean of 3 studies) or vinblastine (mean of 4 studies) for 48 hr.

We hypothesized that increasing mtCa^++^ levels would increase oxidative stress enhancing mitochondrial-induced cell death by cancer therapeutics. BCL-2 inhibitors were studied first since they mediate mitochondrial-induced cell death. ABT-263 (Navitoclax) inhibits BCL-2 and BCL-xL and ABT-199 (Venetoclax) is specific for BCL-2. Cell counts were performed using a ViBlue cell counter, which stains cells with trypan blue to determine cell death. Synergy was determined by calculating the combination index [30]. JL189 combined with ABT-263 synergistically induced cell death in both NSLC and leukemia cell lines (Fig. 5A–B). In total, we found that JL189 combined with ABT-263 synergistically induced cell death in 4 NSCLCs, 2 acute lymphoblastic leukemia (T-ALL) cell lines (Jurkat and DNT-41), 2 acute myelogenous leukemia (AML) cell lines (Kasumi1, THP-1) **(Table S1)**. BMPR2i with JL189 alone and when combined with ABT-263 induced more cell death in leukemia cell lines (Fig. 5B) compared to NSLC cell lines (Fig. 5A), despite using a lower dose of ABT-263. In leukemia cells, JL189 in combination with ABT-199 (Venetoclax) induced synergistic cell death with little response in NSLC cells **(Fig S2)**. The immortalized human embryonic kidney cells (HEK292) were not responsive to the combination of JL189 and ABT-263 (Fig. 5C), suggesting that cancer cells are more susceptible to cell death induced by this combination.

### BMPR2i combined with ABT-263 induces mitochondria-induced cell death.

In leukemia and NSCLC cell lines, the combination of JL189 with ABT-263 after 5 hr induced a much greater increase in the expression of activated caspase-3 fragment and cleavage of PARP-1 compared to each compound alone (Fig. 5D). Jurkat cells exhibited a significant decrease in the mitochondrial membrane potential (MMP) after 2 hr when JL189 was combined with ABT-263 but not with either compound alone (Fig. 5E). The MMP of a mouse hippocampal neuronal cell line (HT-22 cells) did not decrease after treatment with JL189 combined with ABT-263 (Fig. 5F). In H1299 cells, the combination of JL189 and ABT-263 significantly increased MMP compared to each compound alone (Fig. 5G). Hyperpolarization of the mitochondrial can occur when electron transport is dysfunctional. Mitochondrial respiration in H1299 cells was significantly decreased after treatment with the combination of JL189 and ABT-263 compared to that after each treatment alone (Fig. 5H), demonstrating mitochondrial dysfunction. These studies suggest that the combination of JL189 and ABT-263 promotes mitochondrial dysfunction to induce cell death.

### BMPR2i synergistically enhances cell death when combined with microtubule-targeting drugs.

We examined whether BMPR2i synergizes with commonly used chemotherapeutics that target microtubules. When used in combination with JL189, vincristine synergistically induced cell death in the T-ALL, AML, and CML HAP-1 cell lines **(Table S1)**. Synergistic cell death also occurred in K-Ras-mutated NSCLC cell lines treated with JL189 and Taxol **(Table S1)**. To confirm that BMPR2i is required for the observed synergistic cell death, A549 WT and BMPR2 KO cells were examined. Both Taxol and vinblastine induced significantly more cell death in the A549 BMPR2-KO cells than in the A549 WT cells (Fig. 5I).

### BMPR2 inhibition combined with ABT-263 synergistically increases mtCa ^++^ levels.

**Figure 6:**
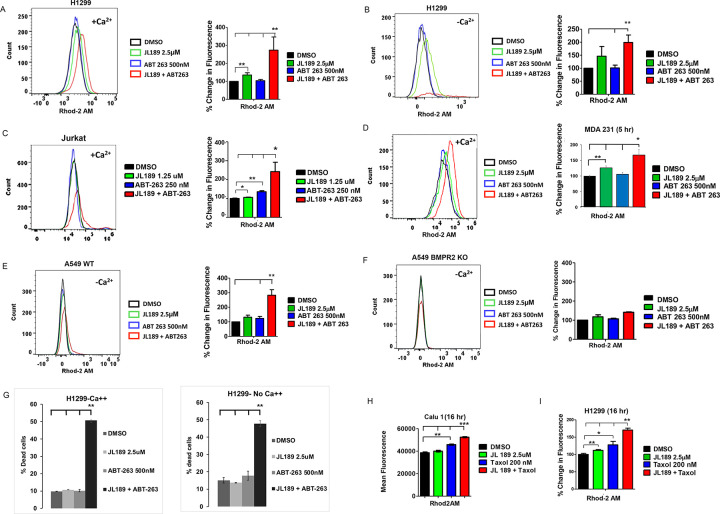
BMPR2i combined with ABT-263, or Taxol synergistically increases mtCa^++^ levels. **(A-F, H, I)** Cells were treated then loaded with Rhod2AM and DAPI and live cells were examined by flow cytometry. **(A,B,C,D,H,I)** Graphs represent the percent change of 4 studies. **(E-F)** Graphs represent the percent change of 2 studies. **(A-C, E-F, H-I)** Cells were treated for 16 hr, except **(D)** which was treated for 5 hr. The cell culture media contained Ca^++^ except for **(B, E-F)** which did not contain Ca^++^ in the cell culture media. **(G)** H1299 cells were treated for 24 hr in cell culture media with or without Ca^++^. The data represents the mean percentage of dead cell of 2 studies.

Ca^++^ enhances mitochondrial respiration by regulating three TCA cycle dehydrogenases, thereby directly controlling ATP synthesis [31]. However, when mtCa^++^ levels become too high, mitochondria-induced cell death is triggered [32, 33]. Since BCL-2/BCL-xL inhibit the influx of Ca^++^ into mitochondria [34], we examined whether the combination of JL189 and ABT-263 synergistically increased mtCa^++^ levels. The cells were loaded with Rhod2AM and DAPI to exclude dead cells from the analysis. Significantly greater mtCa^++^ levels were detected in H1299, A549, Jurkat, and MDA-231 cells treated with the combination of JL189 and ABT-263 than in those treated with each compound alone (Fig. 6A, C, D, E). When calcium was removed from the cell culture medium, the combination of JL189 with ABT-263 still induced the highest mtCa^++^ levels (Fig. 6B, E). A549 BMPR2 KO cells did not show increased mtCa^++^ levels in response to JL189 alone or in combination with ABT-263 (Fig. 6F). Synergistic cell death induced by JL189 combined with ABT-263 also occurred in the presence or absence of Ca^++^ (Fig. 6G). These studies demonstrate significantly increased mtCa^++^ levels induced by BMPR2 inhibition combined with ABT-263, which is associated with cancer cell death. These studies also show that cell death and elevated mtCa^++^ levels are not dependent on the influx of extracellular Ca^++^.

### BMPR2 inhibition combined with Taxol synergistically increases mtCa ^++^ levels.

A synergistic increase in mtCa^++^ also occurred in Calu1 and H1299 cells treated with JL189 combined with Taxol (Fig. 6H, I). MT-targeting agents are not known to increase mtCa^++^ levels. However, MT-targeting agents increase reactive oxygen species (ROS), which can increase mtCa^++^ uptake through the mitochondrial uniporter (MCU) [35]. This raised the question of whether MT-targeting agents and BCL-2 inhibitors increase mtCa^++^ levels by increasing ROS levels.

### BMPR2 inhibition combined with ABT-263 synergistically increases ROS levels, which regulates mtCa ^++^ levels and cell death.

**Figure 7:**
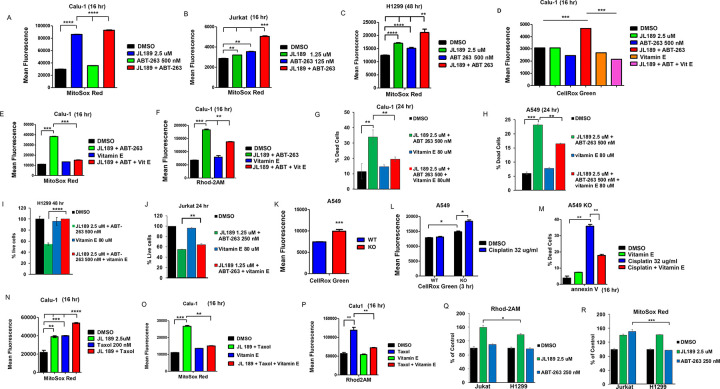
BMPR2i combined with ABT-263 or Taxol synergistically increase mtROS, which regulates mtCa^++^ levels and cell death. **(A-C)** Cells were treated with JL189 and ABT-263 alone or in combination then loaded with MitoSox Red or CellRox Green then analysed by flow cytometry. Cells were treated with JL189 2.5 mM combined with ABT-263 500 nM with and without Vitamin E 80 mM. Cells were then loaded with **(D)** CellRox Green, **(E)** MitoSox Red, or **(F)** Rhod2AM and examined by flow cytometry. Graphs represent the mean of 3–4 studies. **(G-J)** Cells were treated with JL189 + ABT-263 with and without vitamin E 80 mM. The number of dead and live cells determined using Vi-CELL BLU cell viability analyzer. The graphs show the mean of 4 studies reported as the % dead or % live cells. **(K)** Total total O2· - levels of untreated A549 WT and A549 BMPR2 KO cells are represented as the mean of fluorescence of 4 studies. **(L)** A549 WT and A549 BMPR2 KO cells were treated with cisplatin for 3 hr and then loaded with CellRox Green. **(M)** A549 BMPR2 KO cells were treated with cisplatin with or without 80 mM vitamin E then stained with annexin V-stained and apoptotic cells examined by flow cytometry. **(L-M)** Represent the mean of 2 studies. **(N-P)** Calu-1 cells were treated with JL189 2.5 mM and Taxol 200 nM with or without Vitamin E 80 mM for 16 hr and then loaded with **(N-O)** MitoSox Red or **(P)** Rhod2AM and DAPI. **(Q-R)** Jurkat and H1299 were treated with JL189 or ABT-263 for 2h hr then loaded with **(Q)** Rhod2AM or **(R)** MitoSox Red. **(N-O)** The results are represented as the mean fluorescence and **(Q-R)** the % of control of 3 flow cytometry studies.

Increasing mtCa^++^ promotes oxidative phosphorylation, causing a rise in superoxide anion (mtO2• −) levels [36]. MitoSox Red, a fluorescent mitochondrial superoxide indicator, was used to measure mtO2• − levels. JL189 increased mtO2• − levels in Calu-1, Jurkat, and H1299 cells (Fig. 7A–C). When JL189 was combined with ABT-263, mtO2• − levels were significantly greater in Calu1 (Fig. 7A), Jurkat (Fig. 7B), and H1299 (Fig. 7C) cells compared to cells treated with either compound alone. CellRox Green, which measures both mitochondrial and cytosolic O2• levels, also resulted in significantly greater O2• − levels in Calu-1 cells treated with the combination of JL189 and ABT-263 (Fig. 7D).

Increasing free radicals increase mtCa^++^ levels [36], which can amplify the increase in mtCa^++^ and ROS levels [36]. The free radical scavenger vitamin E was used to determine the role of ROS in regulating mtCa^++^ levels and cell death. Vitamin E effectively decreased the increase in total O2•− (Fig. 7D) and mtO2•− (Fig. 7E) levels in cells treated with JL189 combined with ABT-263. Vitamin E also significantly decreased the increase in mtCa^++^ levels in Calu-1 cells (Fig. 7F) and decreased cell death in Calu1–1, A549, H1299, and Jurkat cells induced by JL189 combined with ABT-263 (Fig. 7G–J). These studies show that the combination of JL189 with ABT-263 induces high ROS levels, which further increases mtCa^++^ levels leading to cell death.

### BMPR2 KO increases O2•− levels.

Compared with WT cells, A549 BMPR2-KO cells expressed more total O2•− (Fig. 7K). Compared with WT cells, BMPR2 KO cells exhibited greater increases in O2•− levels when treated with cisplatin (Fig. 7L). Treatment with vitamin E significantly decreased the death of BMPR2-KO cells treated with cisplatin (Fig. 7M). BMPR2 KO studies confirmed that BMPR2i increases O2•− levels. These studies also validate that BMPR2i primes cancer cells for further increases in ROS production when they are challenged with a cancer therapeutic, which induces cell death.

### Taxol increases O2• − levels, leading to increased mtCa ^++^ levels.

We next asked whether the chemotherapeutic Taxol, that used to treat NSLC, also induces an increase in mtROS levels, which regulates an increase in mtCa^++^ levels. Taxol increased mtO2•− levels in Calu1 cells (Fig. 7N). The combination of JL189 and Taxol increased mtO2•− to levels greater than those induced by either compound alone (Fig. 7N). Vitamin E effectively attenuated the increase in mtO2•− induced by JL189 combined with Taxol (Fig. 7O). Vitamin E decreased the increase in mtCa^++^ levels induced by Taxol (Fig. 7P). These studies demonstrate that Taxol and potentially other chemotherapeutic agents increase ROS levels that promote Ca^++^ uptake into the mitochondria. These studies suggest that BMPR2 inhibition synergizes with cancer therapeutics by inducing high ROS levels, which amplifies the influx of calcium into the mitochondria, leading to mtROS and mtCa^++^ overload.

### The increase in mtCa ^++^ and mtROS induced by JL189 and ABT-263 is greater in Jurkat cells compared to H1299 cells.

To better understand why leukemia cells are more sensitive to cell death, we compared Jurkat and H1299 cells using the same dose of JL189 and ABT-263. Because cell death was so high, we used a lower dose of JL189 and ABT-263 leukemia cells compared to the NSLC cell lines. At the same dose, JL189 caused a greater increase in mtCa^++^ levels after 2 hr in Jurkat cells compared to H1299 cells (Fig. 6Q). ABT-263 caused a significantly greater increase in mtO2•levels in Jurkat compared to H1299 cells (Fig. 6R). These data suggest that leukemia cells are more sensitive to BMPR2i induced increase in mtCa^++^ levels and BCL-2 inhibition increase in mtROS levels compared to NSLC cells.

### BMPR2i does not regulate NADPH oxidase to induce cell death.

O2•− can also be produced by NADPH oxidase in the cytosol [36]. NADPH oxidase activity is inhibited by apocynin. Apocynin did not decrease the cell death induced by JL189 combined with ABT-263 in either A549 or Calu1 cells **(Fig S3A-B)**. Glutathione is the major free radical scavenger in a cell. If BMPR2i induces oxidative stress in the cytosol, then depleting glutathione should synergize with JL189. L-Buthionine sulfoximine (BSO) is an inhibitor of g-glutamylcysteine synthetase and depletes glutathione levels. BSO did not increase the degree of cell death induced by JL189 in A549 or Calu1 cells **(Fig S3C-D)**. These findings support our other studies demonstrating that mitochondria are the source of ROS that induces cell death in cells treated with JL189 and ABT-263.

### BMP signaling regulates the influx of Ca ^++^ into the mitochondria induced by ROS.

**Figure 8:**
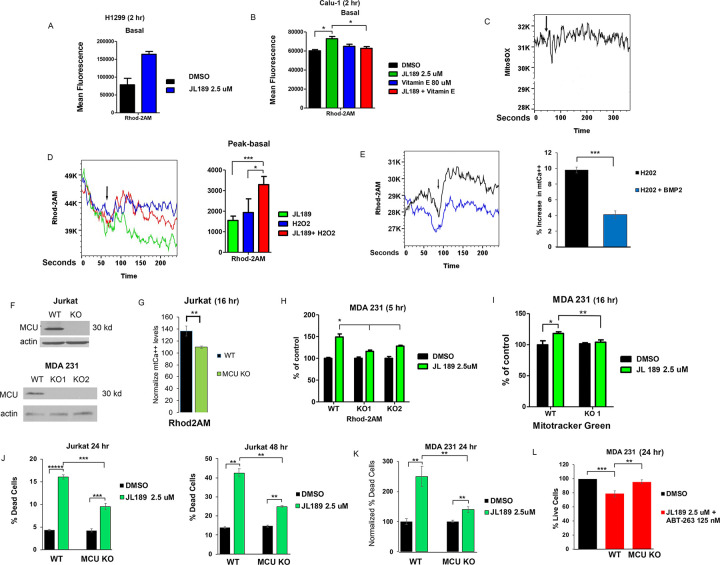
BMPR2 signaling regulates redox sensing of the MCU. **(A-B)** BMPR2i with JL189 increases basal mtCa^++^ levels that is inhibited with vitamin E. The graphs show the mean Rhod2AM fluorescence of 4 studies cells treated for 2 hr. **(C)** Representative study of Calu-1 cells loaded with MitSox Red then treated with JL189 2.5 mM and analyzed by flow cytometry for 3 minutes. **(D)** Calu-1 cells loaded with Rhod-2AM in Ca^++^ free media, were treated with JL189 2.5 mM, hydrogen peroxide (H2O2), or the combination of JL189 and H2O2 and then analyzed by flow cytometry for 3 minutes. Arrows mark the time of treatment. The graph represents the mean of the peak minus the basal values of 4 experiments. **(E)** Calu1 cells loaded with Rhod-2AM were treated with H2O2 500 mM or H202 500 mM pretreated with BMP2 ligand 20 ng/ml for 45 minutes and then analyzed by flow cytometry for 3 minutes. The graph represents the mean percent increase from the basal to peak values of 3 experiments. Studies suggest that BMPR2 signaling inhibits ROS- induced uptake of calcium into the mitochondria. **(F)** Western blot analysis of Jurkat and MDA 231 WT and MCU KO cells. **(G-H)** Jurkat and MD1 231 WT and MCU KO cells were treated with JL189 2.5 mM or vehicle for 16 or 5 hr. **(G)** The data represents the mean fluorescence intensity of Rhod2AM+/DAPI- cells of 4 studies normalized to control. **(H)** Data represent the mean Rhod2AM fluorescence intensity of 2 studies presented as percent change from control. **(I)** MDA 231 WT and MCU KO cells were treated with JL189 for 16 hr then loaded with MitoTracker Green and analyzed by flow cytometry. The graph shows with mean fluorescence of 6 studies presented as the percent change from control. **(J-K)** Cell counts of **(J)** Jurkat and **(K)** MDA-231 WT and MCU KO cells treated with **(J)** JL189 for 24 and 48 hr. The graph represents the mean percentage of dead cells of at least 4 studies. **(K)** Cells were treated for 16–24 hr and % increase in dead cell normalized to control. Data represents the mean of 5 studies. **(L)** Cell counts of MDA 231 WT and MCU KO cells treated with JL189 combined with ABT-263 for 24 hr. The data are presented as the mean percentage of live cells in 4 studies normalized to the control.

After 2 hr of treatment with JL189, the baseline mtCa^++^ levels remained elevated (Fig. 8A–B). When cells were pretreated with vitamin E, JL189 did not increase basal mtCa^++^ levels (Fig. 8B). These data suggest that the sustained increase in mtCa^++^ levels after treatment with JL189 is mediated by an increase in ROS levels. JL189 did not increase mtO2• levels during the first 3 minutes after treatment in Calu1 cells (Fig. 8C), suggesting that the initial increase in mtCa^++^ levels is not dependent on an increase in ROS. Hydrogen peroxide (H2O2) rapidly increased mtCa^++^ levels, which were enhanced by JL189 in Calu1 cells (Fig. 8D). Conversely, the addition of BMP2 ligand decreased the increase in mtCa^++^ levels induced by H2O2 (Fig. 8E). These studies suggest that BMP signaling regulates the influx of calcium into the mitochondria induced by ROS.

### BMPR2 inhibition does not regulate lysosomal or endoplasmic reticulum (ER) Ca ^++^ stores.

Lysosomes and the ER directly transfer Ca^++^ into mitochondria through voltage-dependent anion channels (VDAC) [37]. Under Ca^++^-free conditions, if JL189 induced the transfer of Ca^++^ from either the ER or lysosomes, the remaining stored Ca^++^ would be decreased. Thapsigargin was used to deplete the remaining ER Ca^++^ stores. ML-SA1, which activates the lysosome efflux receptor TRPML-1 [38], was used to deplete lysosomal Ca^++^ stores. H1299 cells treated with JL189 for 16 hr in media without Ca^++^ did not significantly deplete ER or lysosomal Ca^++^ stores **(Fig S4A-B)**. Chronic myelogenous leukemia HAP-1 VDAC1 KO cells have a 14 bp deletion in exon 6. VDAC1 knockout did not significantly attenuate the increase in mtCa^++^ levels after treatment with JL189 for 2 hr or 4 hr **(Fig S5A-B)**. VDAC1 KO did not affect the mitochondrial mass or cell growth of cells treated with JL189 **(Fig S5C-D)**. These studies suggest that BMPR2i does not regulate the ER or lysosomes to increase mtCa^++^ levels.

### BMPR2 inhibition-induced increases in mtCa ^++^ levels, mitochondrial mass, and cell death are dependent on the mitochondrial uniporter (MCU).

MCU regulates the rapid entry of cytosolic Ca^++^ into the mitochondrial matrix [39]. To test whether BMP signaling mediates Ca^++^ uptake through MCU, we utilized Jurkat and MDA 231 cells with MCU KO via CRISPR-Cas9 [19, 20]. Western blot analysis confirmed the KO of MCU (Fig. 8F). Compared with control cells, Jurkat WT cells treated with JL189 for 16 hr had a 37% greater increase in mtCa^++^ levels (Fig. 8G). Compared with control cells, Jurkat MCU KO cells exhibited only a 10% greater increase in mtCa^++^ (Fig. 8G). JL189 caused a 49% increase in mtCa^++^ levels in MDA 231 WT cells compared with a 16% increase in mtCa^++^ levels in MDA 231 MCU KO clone 1 cells and a 28% increase in mtCa^++^ levels in clone 2 cells after 5 hr (Fig. 8H). These studies suggest that the influx of Ca^++^ into the mitochondria induced by BMPR2i is regulated through the MCU.

JL189 increased the fluorescence intensity of MitoTracker Green by 18% in the MDA-231 WT cells and increased it by only 4% in the MDA-231 MCU KO cells (Fig. 8I). JL189-induced cell death was significantly greater in Jurkat WT cells than in MCU KO cells after 24 and 48 hr (Fig. 8J). JL189 induced more cell death in the MDA 231 WT cells than in the MCU KO cells (Fig. 8K). Compared with that in WT cells, growth suppression induced by the combination of JL189 and ABT-263 was partially suppressed in MDA-231 MCU KO cells (Fig. 8L). These data suggest that the increase in mitochondrial bioenergetics and cell death induced by BMPR2i are mediated by an increase in the influx of Ca^++^ through the MCU.

### Treatment with JL189 combined with ABT-263 synergistically decreased the growth of lung tumor xenografts in mice.

**Figure 9:**
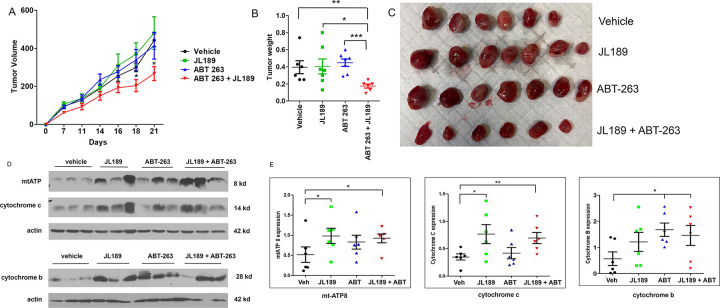
JL189 enhances mitochondrial bioenergetics and synergistically decreases the growth of tumor xenografts when combined with ABT-263. **(A)** H1299 cells (2 million) were injected subcutaneously into the flanks of Balbc nude mice. After 12 days, the mice were then treated with vehicle, JL189 30 mg/kg twice daily intraperitoneally (IP), ABT-263 (Navitoclax) 12 mg/kg IP once daily, alone or in combination for 3 weeks. There were 6 mice per group. **(A)** Tumor volume was measured twice weekly (L x W). **(B)** Tumor weights were determined on day 23. **(C)** Photograph of the tumors. **(D)** Western blot analysis of tumors treated with control, JL189, ABT-263, or JL189 + ABT-263 for 3 weeks. **(E)** Expression was normalized to that of actin of the 6 tumors in each group.

Since JL189 and ABT-263 when used alone induce minimal cell death in H1299 cells in vitro, we examined synergy in tumor xenografts in mice using H1299 cells. JL189 or ABT-263 alone had no effect on tumor growth of H1299 tumor xenografts. There was an approximately a 50% greater reduction in the tumor weight in mice treated with the combination of JL189 and ABT-263 compared to mice treated with vehicle or each compound alone (Fig. 9A–C). Pharmacokinetic studies demonstrated that JL189 has a serum half-life of only 60 minutes in mice **(Table S2)**. Even with twice daily dosing, the therapeutic window is estimated to be only 4 hr. This study supports that the combination of JL189 combined ABT-263 induced synergy in lung tumor xenografts despite a short therapeutic window in mice.

### BMPR2 inhibition enhances mitochondrial bioenergetics in lung tumor xenografts in mice.

Compared with those treated with vehicle, tumors treated with JL189 alone or in combination with ABT-263 had significantly greater expression of mtATP and cytochrome c (Fig. 9D–E). The combination of JL189 and ABT-263 had significantly higher expression cytochrome b that trended toward significance with JL189 alone (Fig. 9D–E). mtATP and cytochrome b are transcribed from mitochondrial DNA [25, 40]. These data suggest that BMPR2 inhibition increases mitochondrial bioenergetics in lung tumor xenografts.

## Discussion

Although BMP signaling is the oldest conserved signaling pathways in metazoans, its role in regulating energy homeostasis has only recently been realized [10]. Our prior reports suggested that BMP signaling negatively affects energy homeostasis, which may have implications in cancer and other age-related diseases [10]. In this study, we show that BMPR2i improves mitochondrial bioenergetics, as demonstrated by an increase in TCA cycle intermediates, oxidative phosphorylation, and mitochondrial mass. An improvement in mitochondrial bioenergetics was shown not only in cancer cells but also in normal mouse hippocampal neurons and in *C. elegans*, suggesting that the regulation of energy homeostasis by BMPR2 is conserved.

The regulation of ROS levels is a conserved mechanism determining stem cell self-renewal and the initiation of differentiation [41]. During self-renewal, stem cells have low respiratory capacity and are highly dependent on glycolysis, similar to the Warburg effect in cancer cells [41, 42]. Although BMP signaling is a critical regulator of self-renewal and cell fate decisions, it has not been previously shown to regulate ROS levels. Interestingly, in cancer cells, BMP signaling regulates mitochondrial ROS and Ca^++^ levels, and the regulation of mtCa^++^ levels is conserved in *C. elegans.* Our studies suggest that BMPR2i regulates MCU to increase mtCa^++^ levels. The increase in mtCa^++^ levels regulated mitochondrial mass, and some of the cell death induced by BMPR2i alone.

Since cancer cells are already under oxidative stress, they are more susceptible to additional increases in ROS levels [43–45]. BMPR2i alone may have beneficial effects on mitochondrial bioenergetics in normal cells as suggested in HT-22 cells. However, in cancer cells, the increased oxidative stress induced by BMPR2i appears to prime cells for cell death. Our studies suggest that BMPR2i enhances then influx of calcium into the mitochondria induced by mtROS produced by cancer therapeutics leading the mtROS and mtCa^++^ overload and cell death. ROS and mtCa^++^ are essential for mitochondrial function, but when they are too high, as in our studies, cell death is induced through conserved apoptotic and nonapoptotic mitochondria-induced cell death pathways [36, 46]. Interestingly, synergistically cell death occurred in cell lines with different genetic mutations. These studies suggest that BMPR2i mediated cell death pathways are not dependent on a specific genetic mutation.

Although the comparison between leukemia and NSLC was not complete, we provide evidence that BMPR2i improves some bioenergetic properties in both leukemia and NSLC cells, which involves the influx of Ca^++^ into the mitochondria. The mitochondria in cancer cells often reprogram metabolic pathways to promote survival and chemoresistance. Adaptive mechanisms include mitochondrial trafficking, Ca^++^ transfer, ROS signaling, mtDNA synthesis, and mitochondrial fission [47]. This has led to several approaches to limit adaptive changes that include targeting mitochondrial complexes (I-V), TCA cycle, redox balance, and metabolic pathways [47]. Our studies suggest the BMPR2i regulates redox sensing of the MCU to alter the balance of ROS and mtCa^++^ levels when combined with certain cancer therapeutics. The mechanism by which BMPR2i regulates the MCU needs to be elucidated to better understand how to implement this strategy to treat patient.

Our studies show that BMPR2i with J189 induced higher levels of mtCa^++^ levels and cell death in leukemia cell lines compared to NSLC cell lines. JL189 combined with either ABT-263 (navitoclax) or ABT-199 (venetoclax) induced synergistic cell death in AML and T-ALL leukemia cell lines that was much greater compared to NSLC cell lines. Venetoclax combined with a hypomethylating agent is the first line treatment for AML patients over 75 and for patients who cannot tolerate intensive chemotherapy. The prognosis with the combination of venetoclax and hypomethylating agent is poor with a median survival of only 14.7 months. Importantly, there is no effective treatment of AML with p53 mutation or mixed lineage leukemia (MLL) translocations [48, 49]. Kasumi-1 cells have a p53 mutation and THP-1 cells have a p53 and MLL-AF9 translocation. We show that Kasumi-1 and THP-1 are very responsive to JL189 alone and in combination with venetoclax, suggesting that a BMPR2 inhibitor could be effective in treating AML.

## Conclusions

Our studies suggest that BMPR2i regulates the MCU to increase mitochondrial Ca^++^ levels, which mediate mitochondrial mass and cell death. BMPR2i enhances the influx of Ca^++^ into the mitochondria induced by ROS. When BMPR2i is combined with chemotherapeutics it increases mtCa^++^ and mtROS levels inducing a metabolic switch that initiates conserved mitochondrial cell death pathways. BMPR2i represents a novel approach to induce synergistic cell death by regulating mitochondrial Ca^++^ and ROS levels. Our studies support the continued development of BMPR2 inhibitors into a drug and their evaluation in patients with AML.

## Abbreviations

BMP: Bone morphogenetic protein
BMPR2: Bone morphogenetic protein type 2 receptor
BMPR2i: Bone morphogenetic protein type 2 receptor inhibition
ID1: Bone morphogenetic protein type 2 receptor
XIAP: X-linked inhibitor of apoptosis
TAKLs: TGFß activated kinases
MTs: microtubule
AMPK: AMP-activated kinase
mtCa^++^: mitochondrial calcium
MMP: mitochondrial membrane potential
TCA: tricarboxylic acid cycle
MCU: mitochondrial uniporter
ROS: reactive oxygen species
KO: Knockout
WT: Wild type
C elegans: Caenorhabditis elegans
O2• −: Super oxide anion
BSO: L-Buthionine sulfoximine
H2O2: Hydrogen peroxide
ER: endoplasmic reticulum
VDAC: Voltage-dependent anion channel
OXY: oxidative phosphorylation
AIF: apoptosis-inducing factor
NSCLC: Non-small cell lung carcinoma
T-ALL: T-cell acute lymphoblastic leukemia
AML: Acute myelogenous leukemia
CI: Combination index
